# 4H-SiC MOSFET Threshold Voltage Instability Evaluated via Pulsed High-Temperature Reverse Bias and Negative Gate Bias Stresses

**DOI:** 10.3390/ma17081908

**Published:** 2024-04-20

**Authors:** Laura Anoldo, Edoardo Zanetti, Walter Coco, Alfio Russo, Patrick Fiorenza, Fabrizio Roccaforte

**Affiliations:** 1 STMicroelectronics, Stradale Primosole, 50, 95125 Catania, Italy; 2 Consiglio Nazionale delle Ricerche-Istituto per la Microelettronica e Microsistemi (CNR-IMM), Strada VIII 5, 95121 Catania, Italy

**Keywords:** pMOSFET, 4H-SiC, HTGB, HTRB, pulsed stress, threshold voltage instability

## Abstract

This paper presents a reliability study of a conventional 650 V SiC planar MOSFET subjected to pulsed HTRB (High-Temperature Reverse Bias) stress and negative HTGB (High-Temperature Gate Bias) stress defined by a TCAD static simulation showing the electric field distribution across the SiC/SiO_2_ interface. The instability of several electrical parameters was monitored and their drift analyses were investigated. Moreover, the shift of the onset of the Fowler–Nordheim gate injection current under stress conditions provided a reliable method to quantify the trapped charge inside the gate oxide bulk, and it allowed us to determine the real stress conditions. Moreover, it has been demonstrated from the cross-correlation, the TCAD simulation, and the experimental ΔV_th_ and ΔV_FN_ variation that HTGB stress is more severe compared to HTRB. In fact, HTGB showed a 15% variation in both ΔV_th_ and ΔV_FN_, while HTRB showed only a 4% variation in both ΔV_th_ and ΔV_FN_. The physical explanation was attributed to the accelerated degradation of the gate insulator in proximity to the source region under HTGB configuration.

## 1. Introduction

Due to its superior physical properties compared to silicon (Si), such as a wide band-gap of about 3.2 eV, a critical electric field, and high thermal conductivity, Silicon carbide (4H-SiC), which is a wide-band gap (WBG) semiconductor material, is becoming the emerging candidate for automotive applications, making power transistors with a high power density module featuring high blocking voltage and ultra-low conduction resistance [1,2,3,4,5]. In this context, High-Temperature Reverse Bias (HTRB) and High-Temperature Gate Bias (HTGB) are routinely performed product qualification tests for PowerMOSFET manufacturing [6,7,8,9,10,11,12,13,14]. In particular, the HTGB test is designed to electrically stress the gate insulator by applying a DC bias voltage at a high temperature, detecting any drift in electrical parameters caused by a significant number of charge traps at and near the SiC/SiO_2_ interface, and bulk oxide traps generated as the oxide has been deposed. The HTRB test aims to monitor the drain terminal leakage current of the devices under High-Temperature Reverse Bias conditions over a period of time. HTRB combines electrical and thermal stress; this test can be used to check junction integrity, crystal defects, and ionic-contamination level, which can reveal device weaknesses or degradation effects in the field depletion structures at device edge termination and in surface passivation. The aim of this work is to compare the effects of the, namely, similar—in terms of the electric field across the insulator layer—negative gate bias stress (HTGB) and drain bias stress (HTRB) on PowerMOSFET, respectively. In this context, TCAD simulations were used to select the experimental conditions to obtain a fair comparison of the two methods. In fact, a comparison of the results obtained on PowerMOSFET aging under both HTRB and HTGB, leading to a different drift or degradation of the threshold voltage (V_th_) and to the onset of the Fowler–Nordheim gate conduction (V_FN_), can be fundamental to refine the electrical characterization protocols for device manufacturers [15,16,17,18,19,20,21]. This comparison is of fundamental relevance for device manufacturing and to define the route to “zero-failure” of the field of device under its “mission-profile”.

## 2. Materials and Methods

**Figure 1:**
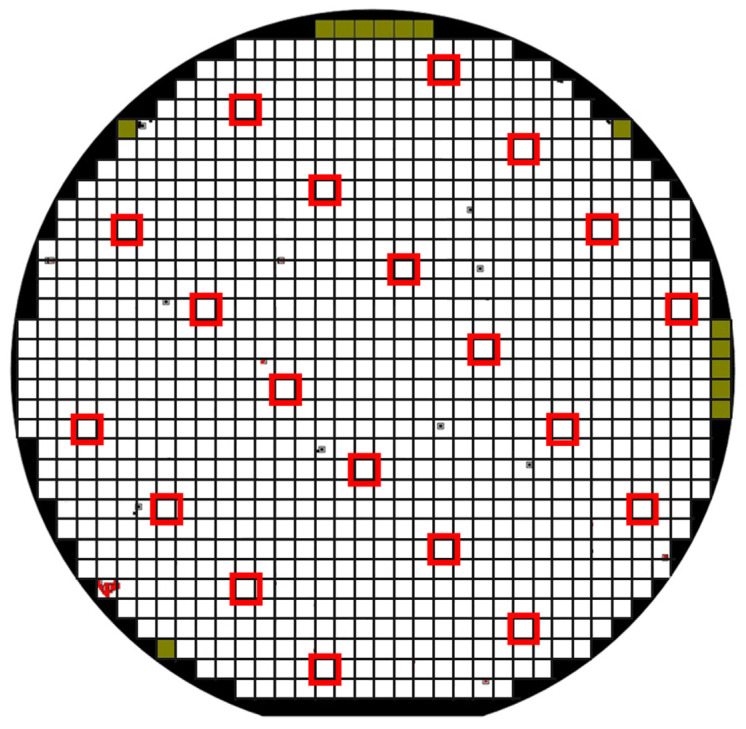
Defectivity maps at epitaxial inspection level. Red: The tested devices that are defect-free and good in the final test.

Several 4H-SiC wafers containing vertical PowerMOSFET transistors were investigated in this work. MOSFETs are fabricated on 150 mm wafers on an n-type (0001) 4H-SiC 180 μm thick substrate with a nitrogen-doping concentration of 4–5 × 10^18^ cm^−3^ and resistivity of 0.012–0.025 Ω·cm. The n-type drift epitaxial layer is 12 μm thick with a nitrogen-doping concentration of 8 × 10^15^ cm^−3^ and is grown by chemical vapor deposition (CVD) in a warm wall multi-wafer reactor. The gate insulation layer is a 50 nm thick deposited oxide layer through a Low-Pressure Chemical Vapor Deposition (LPCVD) process followed by a state-of-the-art NO-based post-oxide deposition annealing [22]. The semiconductor materials were characterized at the beginning of the device fabrication process using microscopic techniques in order to select those devices without any visible epitaxial defect that may affect the device’s reliability [12,23]. Firstly, the optical inspection at the surface of the epitaxial layer is carried out with Candela 8520 using a KLA-Tencor equipment scan which allows us to detect surface defects such as droplets, carrots, triangles, micro-pits, etc. Another optical inspection is performed, using a KLA Altair inspection microscope, after the first photo-lithography step, which defines the pitch of the device, that is, the width, distance, and PowerMOSFET edges. Then, an accurate selection of good devices normally intended for sale is carried out, superimposing defectivity maps, shown in Figure 1, and EWS (Electrical Wafer Sorting). In particular, the EWS is used to select devices distributed on the whole wafer surface, as schematically described in Figure 1 by red squares avoiding “dotted” devices spotted as defect-containing devices by optical inspection performed before and during device fabrication processing.

## 3. Device and Stress Procedure Description

**Figure 2:**
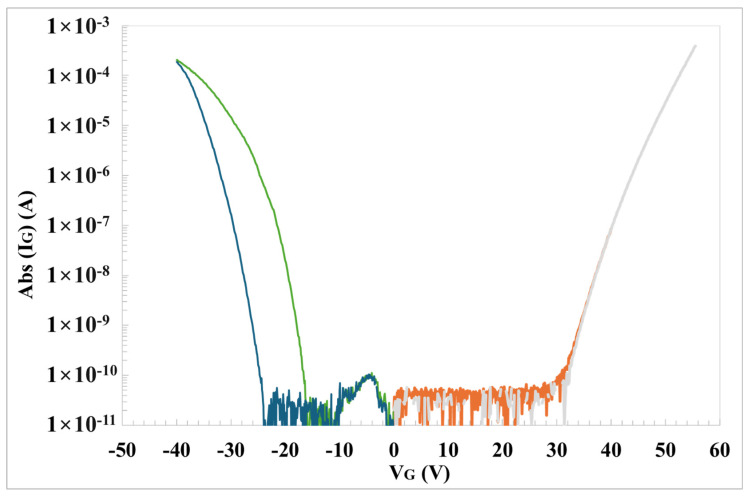
I_G_-V_G_ characteristics of a 500 Å gate oxide on 650 V SiC PowerMOSFET under two consecutive positive (orange and grey) and negative (green and blue) bias sweeps at 200 °C.

A preliminary electrical characterization of the gate oxide and the channel leakage current under reverse gate bias was performed, and it aimed to obtain the onset of the Fowler–Nordheim (FN) tunneling conduction and to define input elements for the TCAD numerical simulation. This iteration is used to define the stress conditions to keep the “real oxide electric field” and the insulator similar, as much as possible, in both HTGB and HTRB conditions. In fact, FN tunneling current it is used to univocally estimate the oxide electric field, the effective thickness and the active semiconductor doping concentration [24,25,26]. Furthermore, both HTGB and HTRB conditions induce different electric field distributions across the MOSFET structure and they can be complementary used to understand which are the more robust and weak components of the elementary cell. In fact, as has been demonstrated previously by Fiorenza et al. [27], Fowler–Nordheim gate conduction can be used as reliable feedback on the control of the oxide electric field. In particular, Figure 2 shows the gate current characteristics (I_G_-V_G_) repeated several times, both on the positive and negative polarization region, and used to define the steady onset of the Fowler–Nordheim (FN) injection current and to determine gate current onset and effective oxide thickness [19,25,28]. Figure 2 shows the I_G_-V_G_ sweeps collected twice consecutively, both on positive and negative polarizations. It can be noticed that in the negative branch of the I_G_-V_G_ (Figure 2), the two consecutive curves do not overlap after repeated negative bias sweeps. This has been demonstrated previously [24] as a manifestation of transient trapping phenomena occurring at the SiO_2_/4H-SiC gate system, resulting in an uncontrolled transient oxide field variation. These measurements are fundamental for giving feedback to the TCAD simulation to set the stress conditions in a well-known oxide field regime.

**Figure 3:**
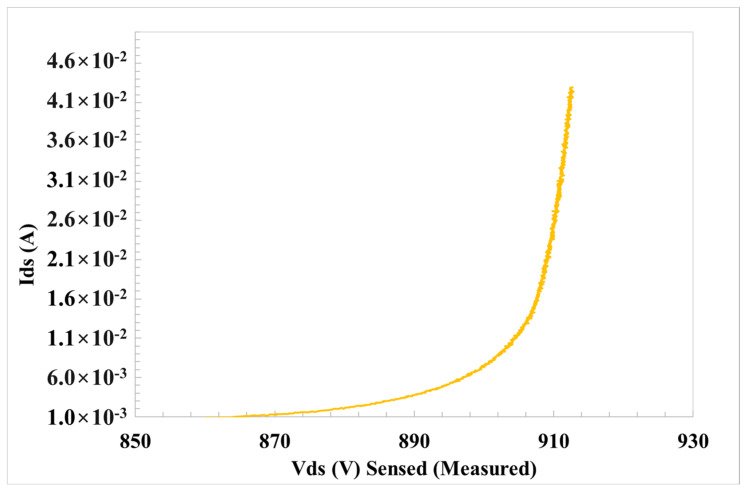
I_D_-V_D_ characteristics under off conditions on a 650 V SiC PowerMOSFET at 200 °C.

On the other hand, Figure 3 shows the I_DS_-V_DS_ characteristics used to determine the operation avalanche breakdown voltage (BV_DSs_) of the device, the semiconductor parameters and to tune the HTRB stress conditions. The occurrence of the avalanche breakdown at a given BV_DSs_ value is fundamental in order to give feedback to the TCAD simulation to set the stress conditions in a well-known semiconductor field regime.

**Figure 4:**
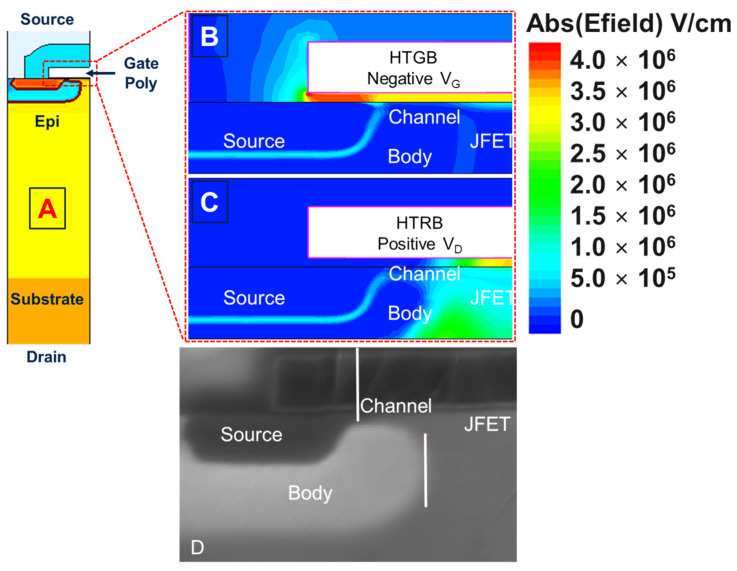
The half-cell structure of PowerMOSFET (**A**). Simulation of the gate oxide field under negative HTGB (**B**) and under HTRB (**C**) conditions. Scanning electron microscopy cross-section image of the MOSFET cell (**D**).

As mentioned in the introduction, the electrical characterization, provided in Figure 2 and Figure 3, was used to understand the experimental conditions in the negative polarization of the gate, such as negative gate bias stress (HTGB) and drain bias stress (HTRB), respectively. TCAD simulations were used to fine-tune the values of V_G_ and V_DS_ in order to obtain similar electric fields in the gate oxide (Figure 4). The schematic of the half-cell structure of SiC Planar MOSFET used in the numerical simulation is presented in Figure 4A. In particular, the static 2D TCAD physical simulations of the gate oxide electric field (E) distribution near the SiC-SiO_2_ interface, under negative HTGB and HTRB conditions, of the 4H-SiC power MOSFET are also shown in Figure 4B and Figure 4C, respectively. Finally, Figure 4D shows the scanning electron microscopy (SEM) performed on the MOSFET elementary cell structure. As can be seen, all the device components (source, body, JFET, etc.) are clearly distinguishable.

**Figure 5:**
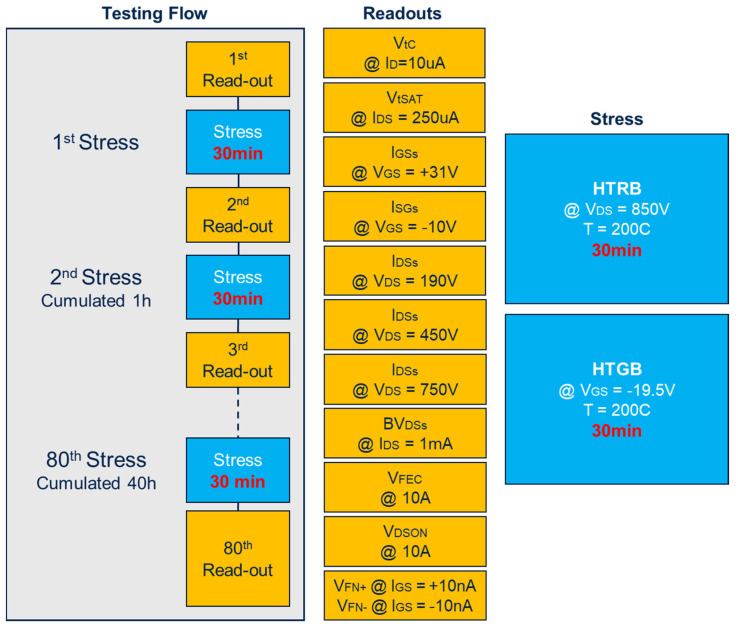
Stress cycling procedure.

A simulation related to HTRB configuration, under the BV_DSs_ condition and limited to I_DSs_ = 1 mA, shows an oxide field maximum value in the JFET region (Figure 4C). On the other hand, the simulation under negative HTGB (Figure 4B), at an equivalent gate oxide field, shows a larger oxide field in the source region compared to the JFET. Hence, although the absolute value of the electric field induced on the gate oxide is the same, under negative HTGB, the field lines at the source/body interface are much higher than in the drift region due to the mismatch in the dielectric constant of the oxide and the semiconductor. Opposite behavior is seen in the case of the HTRB test, where the maximum electric field occurs in the drift region and is practically zero in the source/body region. In this context, it is mandatory to understand the device physics involved in both HTRB and HTGB configurations in order to clarify which particular phenomenon is involved in the different parts of the device degradation. In this scenario, it is fundamental to focus on the impact of these two different stress conditions (having a similar gate oxide field conditions), paying particular attention to the drift of key parameters, represented in Figure 5: the gate and drain terminals’ leakage currents (I_GSs_ and I_DSs_); the gate oxide conduction (V_FN+_ and V_FN−_ namely the Fowler–Nordheim voltage onset by electrons and hole currents coming from the semiconductor); the drain-source breakdown voltage (BV_DSs_); the transistor threshold (V_th_); the threshold with source and drain grounded (V_tSAT_); threshold voltage at 10 µA (V_tC_); the V_DSs_ voltage under conduction (ON-state) condition (V_DSON_); and the body-drain diode forward bias (V_FEC_). The testing protocol is performed using the Keysight Easy Expert platform, as schematically shown in Figure 5.

The characterization flow—schematically depicted in Figure 5—consists of a sequence of stresses and read-outs, i.e., up to 40 h of cumulative stress which equates to 80 stress/read-outs. In each readout, the characteristic parameters of the MOSFET are measured; after the stresses sequence, either HTGB or HTRB are measured. Furthermore, the readout sequence is chosen from the mild perturbation to the more severe perturbation condition.

**Figure 6:**
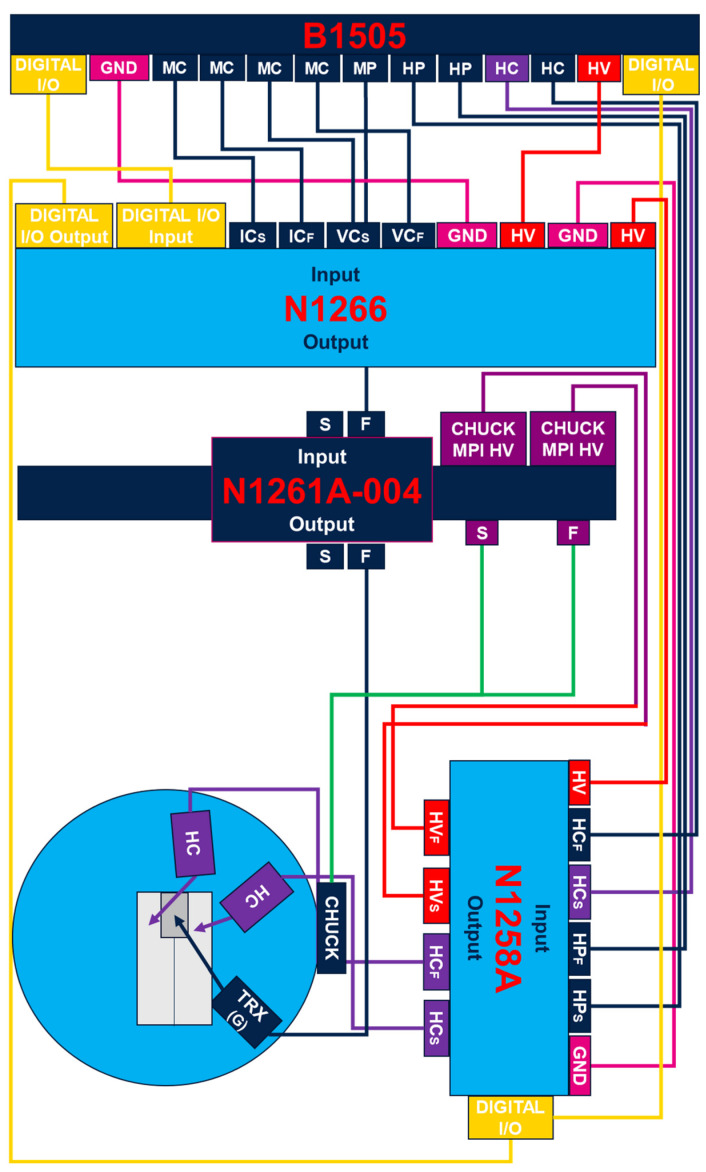
Test setup and system configuration schematic.

The investigation on the drift of the mentioned electrical parameters aims to understand which are the most sensitive parameters to drift under stress conditions. In order to induce significant drift in the selected parameters during stress, the measure/stress cycling routine allows us to control the Keysight B1505 device analyzer (Keysight, Santa Rosa, CA, USA) and the HV MPI-TS2000DP semiautomatic probe-station (MPI Corporation, Zhubei City, Taiwan) to perform wafer level measurements at a temperature of 200 °C. To take advantage of the simulation shown in Figure 4, and to accelerate device degradation, a higher V_DSs_ condition than BV_DSs_ (i.e. V_DSs_ = 890 V/I_DSs_ ~2 mA) for HTRB stress has been chosen, beside an equivalent V_GSs_ stress condition, in terms of a gate oxide field for negative HTBG at V_GSs_ = −19.5 V. The purpose is to put the transistor under an avalanche condition in order to accelerate the stress effects within 40 h. This particular high-voltage/high-current stress-sensing configuration needed an unconventional experimental setup that is worth describing. The enhancement of the power capability until a voltage of ~960 V and leakage current of ~33 mA are reached under pulsed mode [29] is allowed by a Keysight N1266 HVSMU current expander, with interconnection of the so-called Keysight SMU as the MC (Medium Current) and MP (Medium Power) of the B1505. Moreover, in order to switch from a high-voltage to a high-current test, a N1258A Module selector is implemented, as shown in the complete schematic of the connections in Figure 6.

**Figure 7:**
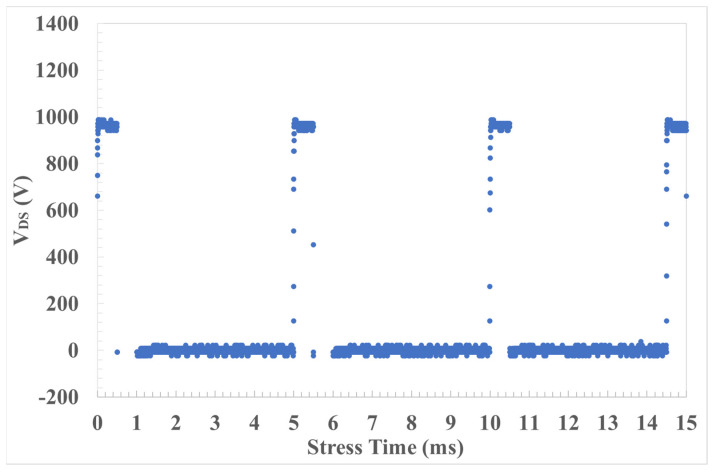
V_D_ stress pulse characterization.

On the other hand, pulsed HTGB and HTRB stress occur close to the operative condition of the real application of the PowerMOSFET subjected to on/off hard switching. Hence, pulsed stress characterization was achieved by placing an oscilloscope with an HV probe in series with the output of the resources. Although the N1266 module allows us to impose a maximum impulse of 1 ms, the resistance of the wiring system, and the consequent misalignment measured between forced voltage and applied voltage, led us to reduce the impulse to 500 µs. Thus, each stress cycle consisted of 1000 pulses with a 5 ms period, repeated 360 times for a total stress time of 1800 s, as shown in Figure 7.

## 4. Data/Results and Discussion

To simplify the graphical representation of drifting parameters under HTRB and HTGB stress, a limited selection of devices under testing has been reported in the figures related to the test. The experimental results have been confirmed on samples collected on different wafers belonging to different production lots. Some representative devices are reported in the following tables labeled as the Dev number belonging to their wafer (Wf) number.

### 4.1. HTRB Test

A preliminary characterization of the devices was performed before the HTRB test by taking into account the different parameters, shown in Figure 5 in orange boxes. Table 1 shows only the parameters, which, after stress, exhibit the most significant drift. It has to be emphasized that V_th_ and V_FN-_ (hole injections from p-body) are affected by the stress. The drift of the other electrical parameters is quite negligible.

### 4.2. HTGB Test

The same preliminary characterization was performed in HTGB stress conditions and the most significant results are, respectively, shown in Table 2 for the first read-out and for drift analysis.

**Figure 8:**
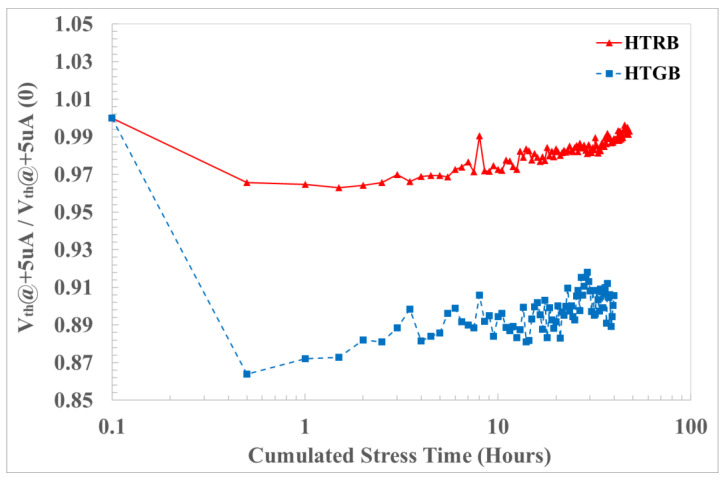
Threshold voltage drift under HTRB at 890 V (red) and HTGB at −19.5 V (blue) stress.

At the end of the characterization protocol, a data comparison of the drift analysis of HTRB-stressed devices and negative equivalent bias under HTGB stress was carried out on V_th_ (Figure 8). The decreasing V_th_ on the blue sample (stressed under HTGB conditions) is higher than that on the red ones (stressed under HTRB conditions). This is usually related to negative charge emission or hole trapping in the gate oxide. It can be noticed that the HTRB caused a variation in the threshold voltage of ΔV_th_ < 4% from the initial value. On the other hand, the HTGB caused a large variation (with respect of the HTRB) in the threshold voltage of ΔV_th_ < 15% from the initial value.

**Figure 9:**
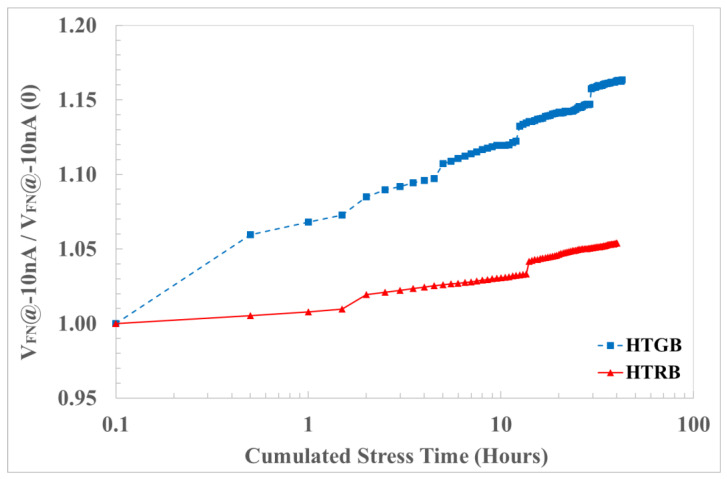
Negative F-N voltage onset drift under HTRB at 890 V (red) and HTGB at −19.5 V (blue) stress.

To confirm these results, and to emphasize the contribution of the charge trapped inside the gate oxide bulk, the V_FN_ drift under positive and negative bias has been put under analysis. The most significant drift, reported in Figure 9, is related to V_FN-_, which is mainly affected by hole injection from the body and electrons from poly. Comparing the results with HTRB equivalent stress, the result is more evident than for V_th_ drift and the V_FN_- shift is higher on the HTGB-stressed samples.

It can be noticed that the HTRB produced a variation in the onset voltage of the Fowler–Nordheim conduction of ΔV_FN_ < 4% from the initial value. On the other hand, the HTGB produced a large variation (with respect of the HTRB) in the threshold voltage ΔV_FN_ of < 15% from the initial value.

This experimental evidence can been understood by looking at the TCAD simulation in Figure 4B,C. In fact, in HTGB (Figure 4B) in the source region, the electric field is larger than that in the field in the JFET region; meanwhile, in HTRB (Figure 4C) in the source region, the electric field is a small fraction of that in the JFET region. Hence, different device degradation can be attributed to the different charge trapping occurring in the gate oxide in the proximity of the source region.

**Figure 10:**
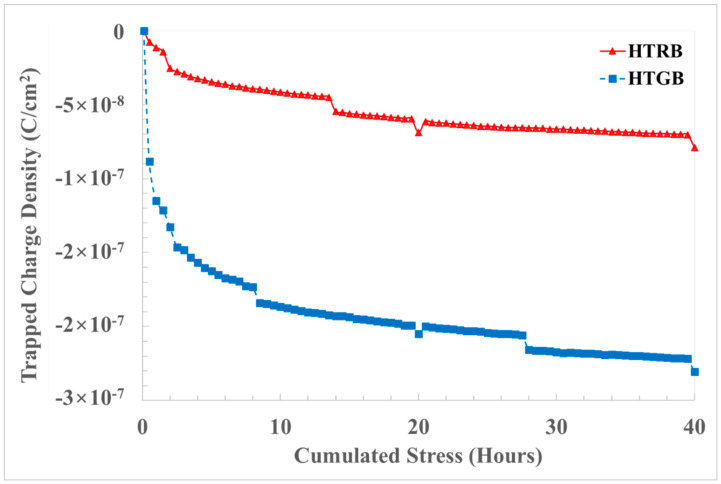
Oxide trapped charge density under HTRB (red) and HTGB (blue) stress.

Finally, we calculated and quantified, by the charge-sensing method [30], the averaged trapped charge density inside the gate oxide. This confirmed the hypothesis of the worst-case effect on gate oxide degradation being that of negative bias gate stress as opposed to HTRB stress, even if the simulated gate field is the same (Figure 10). Negative bias stress causes a negative shift in V_th_, which might be explained by the filling and emptying of near-interfacial oxide traps in the presence of a gate bias stress [31].

## 5. Conclusions

In the field of device reliability, it is fundamental to evaluate which is the experimental procedure that represents the best trade-off between real-world application conditions and an accelerated characterization test to qualify material processing for device fabrication. Therefore, in this paper, a comparison of pulsed HTGB and HTRB stress has been carried out on defect-free 4H-SiC PowerMOSFETs at EWS in order to identify the most sensitive electrical test parameter under different stress conditions. From a wide number of analyzed electrical parameters, V_th_ and V_FN-_ showed higher instability and degradation compared to the others. Moreover, it has been demonstrated from the cross-correlation, the TCAD simulation and the experimental ΔV_th_ and ΔV_FN-_ variation that HTGB stress is more severe compared to HTRB stress. In fact, HTGB showed a 15% variation in both ΔV_th_ and ΔV_FN−_; meanwhile, HTRB showed only a 4% variation in both ΔV_th_ and ΔV_FN-_ after an accumulated stress of 40 h. The physical explanation was attributed to the accelerated degradation of the gate insulator in the proximity of the source region under HTGB configuration.

## Figures and Tables

**Table 1:** Channel- and gate oxide-related parameters before HTRB test and their corresponding drift (I(t)/I(0)) after 40 h of HTRB stress.

	V_th_ (V) @ I_DS_ = 5 µA V_DS_ = 0.1 V	V_FN+_ (V) @ I_GS_ = +10 nA	V_FN-_ (V) @ I_GS_ = −10 nA	BV_DSS_ (V) @ I_DS_ = 1 mA G & S Grounded	
DEV33–WF1	0.523	33.42	−17.30	879.11	Parametric data Before HTRB stress
DEV36-WF1	0.510	33.58	−17.78	881.43	
DEV19-WF2	0.814	36.00	−18.59	853.08	
DEV20-WF2	0.842	35.82	−18.14	853.34	
DEV33–WF1	0.989	1.01	1.07	1.00	Drift analysis After HTRB stress
DEV36-WF1	0.950	1.01	1.05	1.00	
DEV19-WF2	1.014	1.01	1.06	1.00	
DEV20-WF2	1.010	1.01	1.07	1.00	

**Table 2:** Channel- and gate oxide-related parameters before HTRB test and their corresponding drift (I(t)/I(0)) after 40 h HTGB stress.

	V_th_ (V) @ I_DS_ = 5 µA V_DS_ = 0.1 V	V_FN+_ (V) @ I_GS_ = +10 nA	V_FN-_ (V) @ I_GS_ = −10 nA	BV_DSS_ (V) @ I_DS_ = 1 mA G & S Grounded	
DEV34–WF1	0.45	33.68	−17.59	879.82	Parametric data Before HTGB stress
DEV37-WF1	0.51	32.98	−17.38	878.16	
DEV21-WF2	0.89	36.21	−18.41	840.66	
DEV22-WF2	0.82	35.46	−18.55	844.32	
DEV34–WF1	0.96	1.01	1.16	1.01	Drift analysis After HTGB stress
DEV37-WF1	0.91	1.01	1.15	1.01	
DEV21-WF2	0.99	1.01	1.11	1.01	
DEV22-WF2	1.00	1.01	1.10	1.01	

## Data Availability

Data are contained within the article.
